# Dysregulation of lipid metabolism and appearance of slow myofiber-specific isoforms accompany the development of Wooden Breast myopathy in modern broiler chickens

**DOI:** 10.1038/s41598-019-53728-8

**Published:** 2019-11-20

**Authors:** Michael B. Papah, Behnam Abasht

**Affiliations:** Department of Animal and Food Sciences, University of Delaware, Delaware, DE USA

**Keywords:** Gene expression, Fat metabolism

## Abstract

Previous transcriptomic studies have hypothesized the occurrence of slow myofiber-phenotype, and dysregulation of lipid metabolism as being associated with the development of Wooden Breast (WB), a meat quality defect in commercial broiler chickens. To gain a deep understanding of the manifestation and implication of these two biological processes in health and disease states in chickens, cellular and global expression of specific genes related to the respective processes were examined in pectoralis major muscles of modern fast-growing and unselected slow-growing chickens. Using RNA *in situ* hybridization, lipoprotein lipase (LPL) was found to be expressed in endothelial cells of capillaries and small-caliber veins in chickens. RNA-seq analysis revealed upregulation of lipid-related genes in WB-affected chickens at week 3 and downregulation at week 7 of age. On the other hand, cellular localization of slow myofiber-type genes revealed their increased expression in mature myofibers of WB-affected chickens. Similarly, global expression of slow myofiber-type genes showed upregulation in affected chickens at both timepoints. To our knowledge, this is the first study to show the expression of LPL from the vascular endothelium in chickens. This study also confirms the existence of slow myofiber-phenotype and provides mechanistic insights into increased lipid uptake and metabolism in WB disease process.

## Introduction

Wooden Breast (WB) a muscle quality disorder that imparts a firm feel on the pectoral (P.) major muscles of commercial broiler chickens upon palpation, continues to cause significant losses in the poultry industry unabated. Consequently, a number of studies on WB have been conducted to decipher its pathology and pathogenesis^1–4^ as well as molecular dynamics^5–8^ characterizing the disease process in commercial broiler chickens. Histopathological studies of the early phases of the disease have revealed onset of phlebitis targeting smaller-caliber blood vessels and perivascular lipid infiltrations that increase in scope and intensity with disease process over time^1,3^. Similarly, gene expression and biological functional analyses of WB disorder have revealed perturbations in lipid metabolism, remodeling of extracellular matrix and dysregulation of excitation-contraction coupling distinguishing the early stages of the WB myopathy^7^. On the other hand, molecular analysis of WB in the later stages, have showed evidence of myoregeneration and occurrence of fast-to-slow muscle switch intermixed with enhanced fibrosis^6^. Additionally, disturbances in lipid metabolism complexed with oxidative stress as well as compromised carbohydrate metabolism have been observed following metabolomic studies of WB in affected chickens at market age^5^. Based on these previous studies, there appears to be an association of WB disease process with dysregulation of metabolism, especially relating to lipid metabolism, as well as the appearance of slow myofiber phenotype (type I or oxidative myofibers) in the P. major muscles of chickens. It is generally accepted that P. major muscles of chickens are comprised almost entirely of fast-twitch (type IIB) myofibers^9–11^. With fewer mitochondria, capillaries and myoglobin content compared to type I fibers, fast-twitch myofibers rely heavily on glycolysis to meet its metabolic energy demands^12^. It follows, therefore, that P. major muscles in chickens would generally have limited capacity to accomplish oxidative phosphorylation as well as utilization of ß-oxidation of fatty acids for its bioenergetics. In contrast, slow-twitch (type-1 or oxidative) myofibers express slow sarcomeric protein isoforms, have higher mitochondrial content, myoglobin, capillaries as well as lipids, and primarily utilize oxidative phosphorylation for its metabolic functions^12,13^. Based on these observations therefore, the occurrence of lipid dysregulation and emergence of slow-type myofiber genes in the P. major muscles of WB-affected chickens raises pertinent biological questions. 1. What is the cellular localization of some of the genes associated with lipids and slow myofiber-phenotype within the P. major muscle of chickens? 2. Why does the P. major muscle, a predominantly glycolytic muscle tissue exhibit increased expression of lipid metabolism as well as slow-type muscle genes during the development of WB myopathy? 3. What are the possible implications of lipids and slow myofiber proteins in the general muscle metabolism with respect to energy source and utilization?

To answer these questions, we evaluated the cellular localization, as well as global expression of specific genes highly related to lipid metabolism and slow myofiber-specific isofoms during the early and late phases of WB disorder in chickens. This was achieved by utilizing RNA *in situ* hybridization technique to localize the expression of specific genes on the P. major muscles of slow-growing Legacy chickens (not known to develop WB disease), and WB-affected and unaffected Ross birds. We also used RNA-seq expression data from two commercial broiler chicken lines; one at 3 weeks of age (early phase of WB)^7^ and the other at 7 weeks of age (late phase of WB)^6^. From the RNA-seq datasets, we focused on genes related to lipid metabolism and slow-skeletal muscle phenotype. Results from the current study have brought to the forefront new insights into the cellular expression of lipoprotein lipase (LPL) in chickens that was not known before. Additionally, pertinent knowledge showing the relationship between changes in lipid metabolism and occurence of slow myofiber isoforms in the P. major muscles with the development of WB in commercial broiler chickens have been revealed in this study.

## Results

### RNA *in situ* hybridization of lipid-related genes

**Figure 1:**
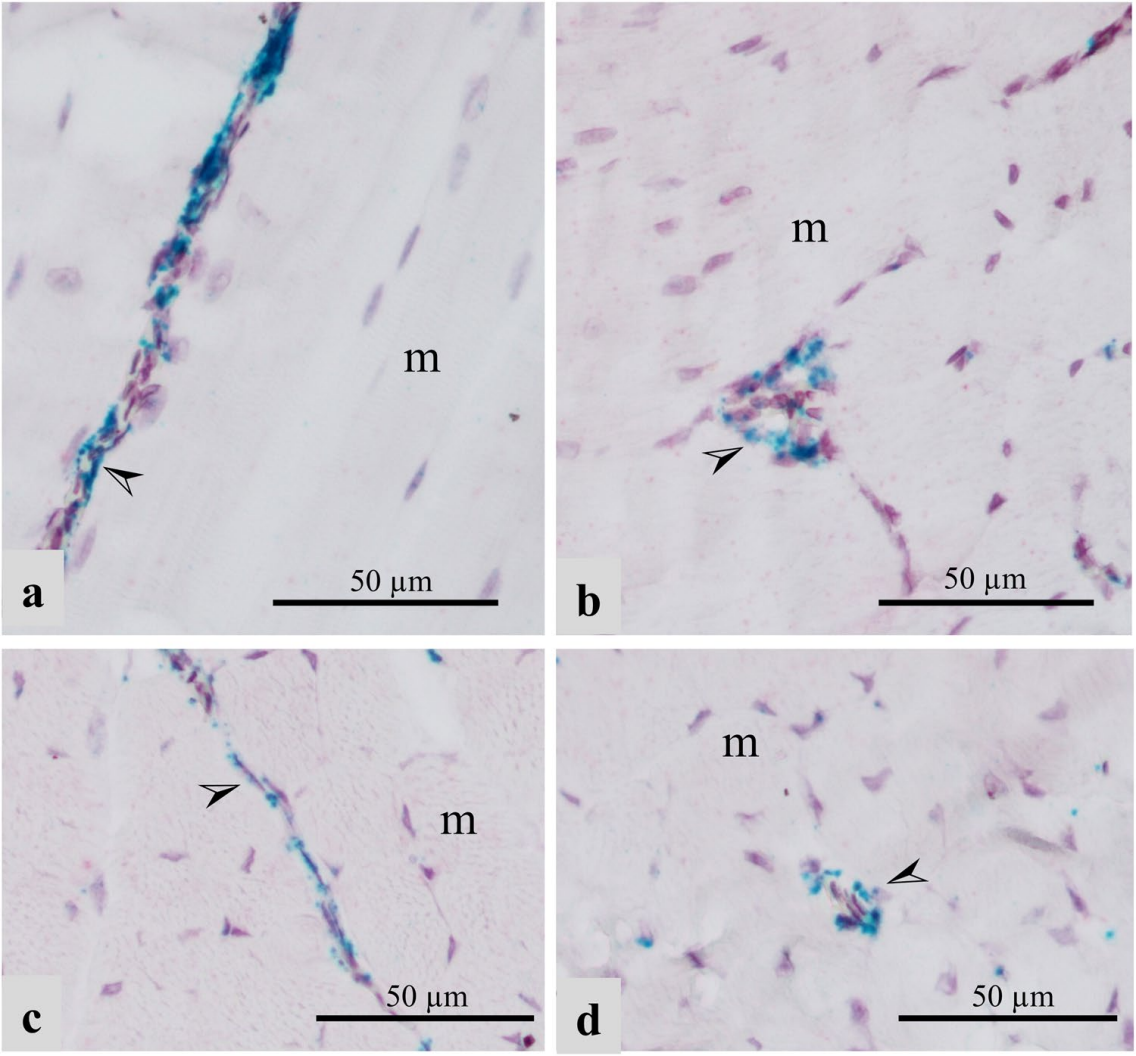
Expression of LPL as revealed by LPL mRNA (green signal) in the vascular endothelium (➢) within the pectoralis major muscles of healthy Legacy (**a** and **b**) and Ross (**c** and **d**) chickens. Blood vessels shown are (**a**: capillaries; **b**: venule) in Legacy chicken, and (**c**: capillary; **d**: venule) in Ross chicken m; myofibers.

**Figure 2:**
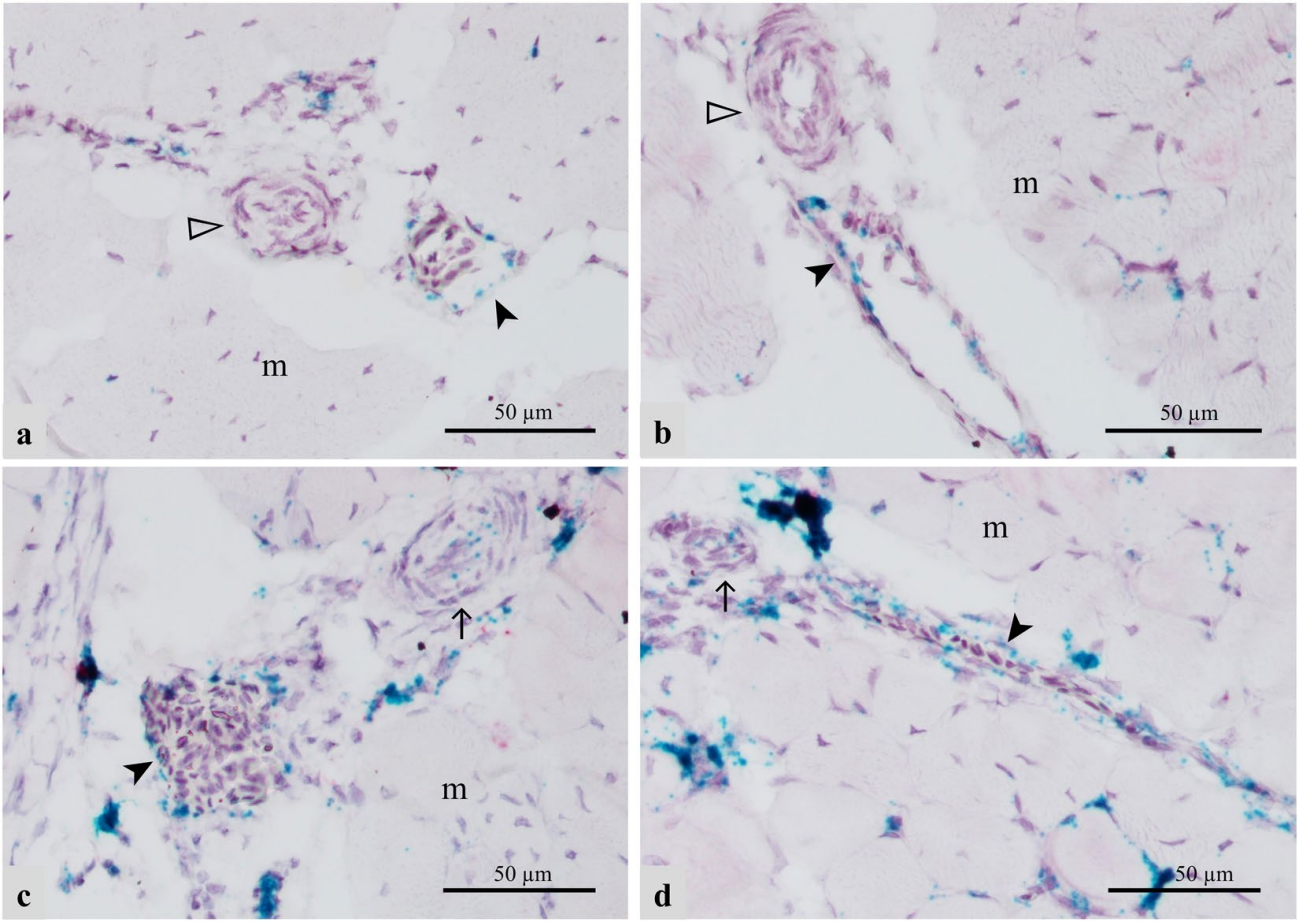
Expression of LPL in form of LPL mRNA (green signal) in the endothelium of veins (➤) in Legacy chicken (**a**) and unaffected Ross chicken (**b**), while arteries (▻) in respective chicken types do not show LPL mRNA signal. Affected Ross chickens (**c**,**d**) with enhanced LPL mRNA signal in veins (➤), while arteries show subtle LPL mRNA signal (↑). Expression of PLIN1 mRNA signal (red signal) in extracellular matrix possibly in developing adipocytes (**c**,**d**) can be seen. m; myofibers.

**Figure 3:**
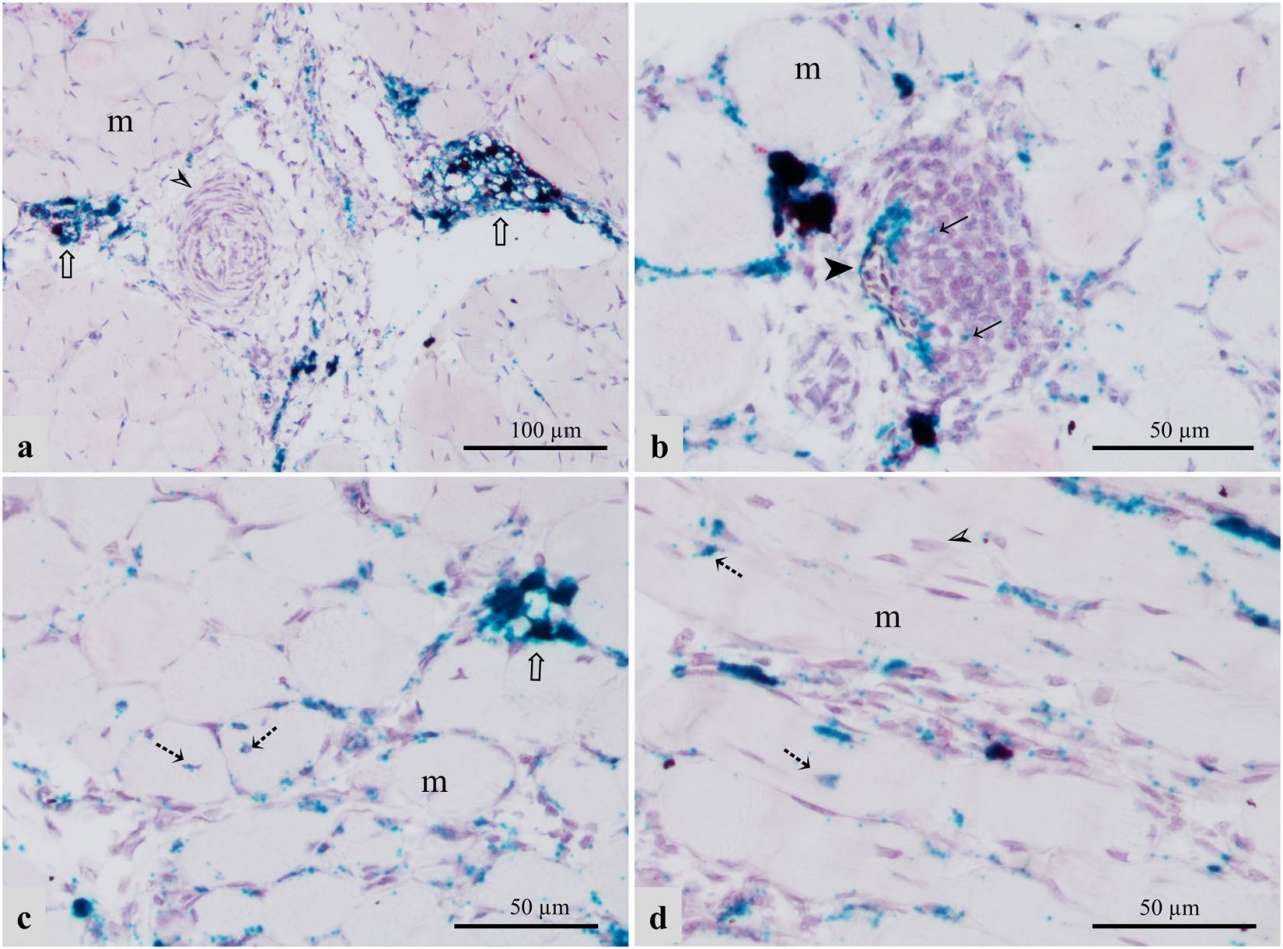
Expression of LPL mRNA (green signal) in the P. major muscle of affected chickens. LPL mRNA signal is enhanced in several sites including developing adipocytes (⇧) in (**a**,**c**); vein undergoing inflammation (➤); capillaries (between myofibers), and some myofibers (⇣) in (**c**,**d**). A few macrophages also expressed LPL (←) in (**b**). PLIN1 mRNA signal (red signal) in developing adipocytes in (**a**) and generally localized in the extracellular matrix in (**b**,**d**). Notice that a large artery in (**a**) (➢) in affected chicken does not express LPL. m; myofibers.

To localize the expression of lipoprotein lipase (LPL) in the P. major muscles in affected and unaffected chickens, we utilized RNA *in situ* hybridization technique. In our study, mRNA signal for LPL appeared to be localized in the endothelial layer of capillaries and small-caliber vasculature within the P. major muscles of both affected and unaffected chickens. In unaffected chickens, LPL mRNA signal was observed in the endothelium of capillaries and venules running between contiguous myofibers in both Legacy (Fig. 1a,b) and Ross birds (Fig. 1c,d). LPL was expressed intermittently along the length of these blood vessels. Similar presentation was also observed in the endothelium lining other small-caliber veins in Legacy chickens (Fig. 2a**)** and unaffected Ross chickens (Fig. 2b). Arteries in unaffected Legacy chickens (Fig. 2a**)** and unaffected Ross chickens (Fig. 2b) did not exhibit LPL mRNA signal. Conversely, affected chickens showed increased signal of LPL mRNA in the endothelial lining of small-caliber veins compared to small-caliber arteries which exhibited lower LPL mRNA signal in their endothelial linings (Fig. 2c,d). The expression of LPL in large arteries in affected chickens was almost nondetectable (Fig. 3a). In addition, the LPL mRNA signal in affected chickens was enhanced in the endothelium of veins undergoing phlebitis, often characterized by intramural infiltrates as well as perivascular cuffing comprising primarily lymphocytic cells and to a lesser extent, macrophages (Fig. 3b). The macrophages found in affected tissues also expressed some LPL as evidenced by the LPL mRNA signal in the said cells (Fig. 3b). Further, there appeared to be increased LPL mRNA signal in developing adipose tissue found in the extracellular matrix (ECM) between muscle bundles of affected chickens, (Fig. 3a,c), capillaries between myofibers, as well as in some myofibers of affected birds (Fig. 3c,d).

Localization of expression of perilipin 1(PLIN1) in the P. major muscles in affected and unaffected chickens was conducted using RNA *in situ* hybridization. The muscles in unaffected chickens (Legacy and Ross) did not show PLIN1 expression. On the contrary, in the muscles of affected chickens, the signal of PLIN1 mRNA was largely observed within the ECM and especially in developing adipose tissue that infiltrated muscle fascicles as well as in proximity to the vasculature (Fig. 2c,d) and (Fig. 3a,b,d). It should be noted that in all the tissues, the expression of PLIN1 was frequently accompanied by the expression of LPL. Besides adipocytes which expressed PLIN1, it was not possible to resolve the identity of other cells expressing PLIN1 gene.

### RNA-seq gene expression of lipid-related genes

**Figure 4:**
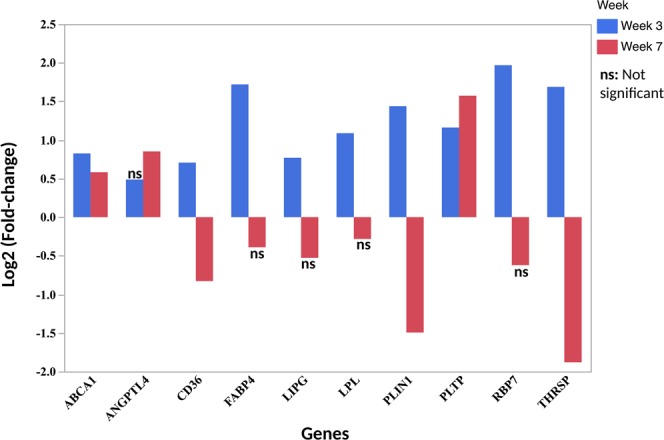
Log2 Fold-change of differentially expressed genes in WB associated with lipid metabolism between affected and unaffected broiler chickens at week 3 and week 7 of age. Positive log2 Fold-change indicate upregulation in affected chickens while negative log2 Fold-change indicate down regulation in affected chickens. Notice that the majority of the genes are upregulated at week 3 and downregulated at week 7 of age. However, the expression of some genes were not statistically different between affected and unaffected chickens and are denoted as **ns** (not significant).

Gene expression data of DE lipid-related genes based on log2 fold-change between affected and unaffected chickens from week 3 of age was compared with those of chickens at week 7 of age. All the lipid-related genes except ANGPTL4, were significant and upregulated in affected chickens compared to unaffected group at week 3 of age (Fig. 4). At week 7 of age, 3 genes namely ABCA1, ANGPTL4 and PLTP were significantly upregulated while CD36, PLIN1, and THRSP were downregulated (Fig. 4). Other genes namely, FABP4, LIPG, LPL and RBP7 were not differentially expressed between affected and unaffected chickens at week 7 of age (Fig. 4).

### RNA *in situ* expression of muscle-related genes

**Figure 5:**
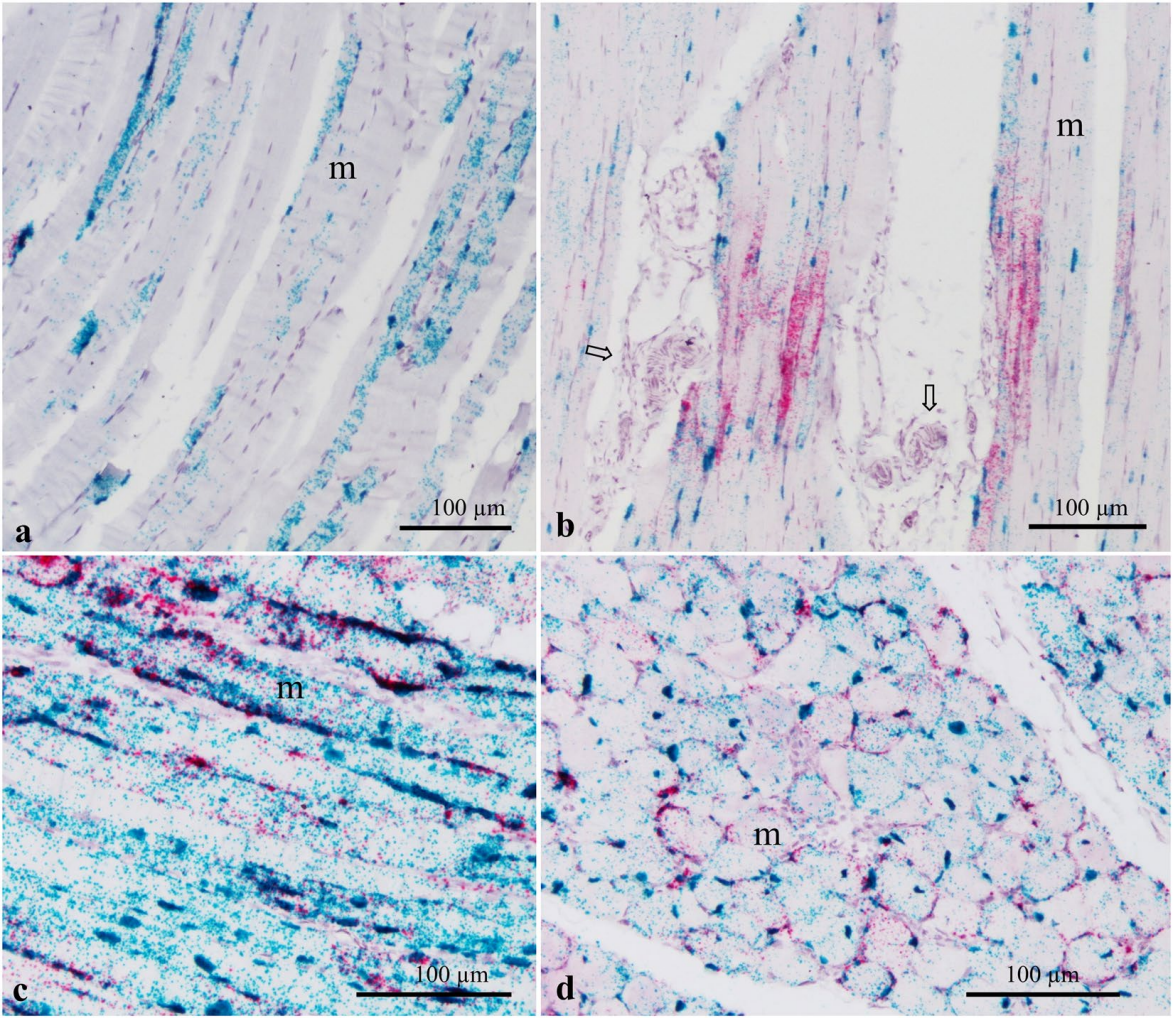
Expression of MYBPC1 (green signal) and CSRP3 (red signal) genes in the P. major muscle of Legacy (**a**), unaffected Ross (**b**) and WB-affected Ross chickens (**c**,**d**). Notice the focal to multifocal expression of MYBPC1 in Legacy and unaffected Ross, and increased MYBPC1 mRNA signal assuming a homogenous distribution in myofibers of affected Ross chickens. CSRP3 expression in Legacy chickens is largely absent, the expression in unaffected Ross chickens occur frequently in portions of myofiber that are contiguous to tissues in the extracellular matrix such as the vasculature (⇧), while in affected chickens, CSRP3 mRNA signal is increased and largely distributed towards the sarcolemma of myofibers (**c**,**d**). Notice that CSRP3 is consistently co-expressed with MYBPC1, but not the other way. m; myofibers.

RNA *in situ* hybridization of MYBPC1 in the P. major muscles of chickens in the present study demonstrated a stage wise expression pattern with respect to WB disease status. In Legacy chickens which are not affected by WB, the signal of MYBPC1 mRNA exhibited intermittent focal distribution along the length of individual myofibers. The areas along the length of myofibers upon which MYBPC1 was expressed appeared to vary widely with no predictable pattern. Similarly, the intensity of the MYBPC1 mRNA signal varied from one region to another along myofibers (Fig. 5a). An almost similar presentation of MYBPC1 mRNA signal was seen in the P. major muscles of unaffected Ross chickens (Fig. 5b). In affected Ross chickens, MYBPC1 mRNA signal was greatly enhanced and was distributed homogenously within the myofibers, covering longer stretches of myofiber lengths (Fig. 5c,d).

Localization of CSRP3 gene was examined using RNA *in situ* hybridization in Legacy, and both affected and unaffected Ross chickens. The mRNA signal of CSRP3 was almost non-existent in the P. major muscles of Legacy chickens (Fig. 5a). On the other hand, CSRP3 mRNA signal was observed in unaffected Ross chickens in a focal manner and in very limited portions of individual myofibers. In particular, the CSRP3 mRNA signal in unaffected Ross chickens was present in portions of myofibers that were adjacent to the vasculature and/or other structures localized within the extracellular matrix (Fig. 5b). In some instances, the signal was stronger in areas where myofibers appeared to be impinged by contiguous vascular tissues (Fig. 5b). In these regions, the expression of MYBPC1 was not altered. In affected chickens, CSRP3 mRNA signal was enhanced assuming a multifocal distribution among fibers (Fig. 5c). Additionally, the CSRP3 mRNA signal in myofibers of affected samples appeared to be highly concentrated along the periphery towards the sarcolemma. In such cases, CSRP3 was always co-expressed with MYBPC1 in myofibers (Fig. 5c,d).

### RNA-seq gene expression of muscle-related genes

**Figure 6:**
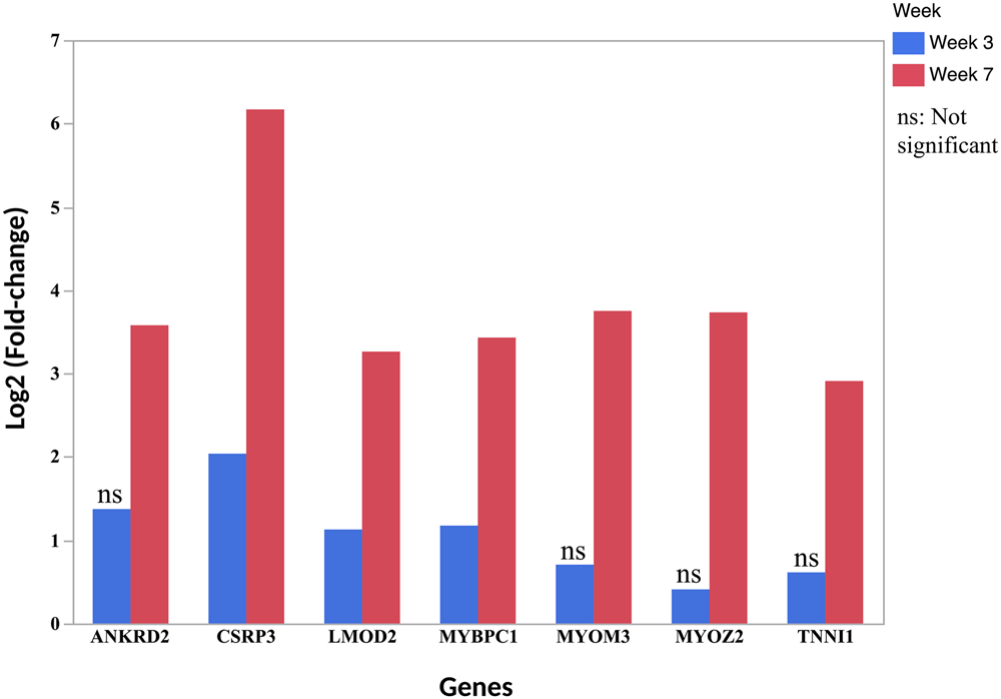
Log2 Fold-change of differentially expressed genes in WB associated with slow-myofiber phenotype between affected and unaffected broiler chickens at week 3 and week 7 of age. Positive log2 Fold-change indicate upregulation in affected chickens while negative log2 Fold-change indicate down regulation in affected chickens. Notice that while all genes are upregulated in affected chickens at week 3 and week 7 of age, the genes in the later age group have appreciably higher fold-change. However, the expression of some genes between affected and unaffected group were not statistically different and are denoted as **ns** (not significant).

Examination of differential expression of all 7 muscle-related genes between WB-affected and unaffected chickens revealed upregulation in the P. major muscle of affected chickens at week 3 and week 7 of age (Fig. 6). However, while all the 7 genes were significantly expressed higher in affected chickens at week 7, only 3 genes were significant at week 3, namely CSRP3, MYBPC1 and LMOD2 (Fig. 6). Further, CSRP3 had the highest fold-change at both week 3 (log2FC 2) and week 7 of age (log2FC 6.2) (Fig. 6**)**.

## Discussion

The present study aimed at demonstrating the evidence as well as mechanistic relationship of lipid dysregulation and occurrence of slow myofiber-phenotype in a typically glycolytic P. major muscle during the development of WB in modern broiler chickens. By using RNA *in situ* hybridization and RNA-seq, it was possible to examine the expression of specific genes as they relate to WB disease process in the early phase (week 3 of age) and late phase (week 7 of age). Some of the genes related to lipid metabolism and myofiber remodeling, and their respective implications in muscle metabolism are discussed.

Lipoprotein lipase (LPL) is the rate-limiting enzyme involved in the hydrolysis of circulating triglycerides (TGs) in chylomicrons, or portomicrons in chickens^14^, and in very low-density lipoproteins (VLDL). This lipid hydrolysis then results in the release of free fatty acids, monoglycerides and other chylomicron/portomicron remnants for use by subjacent tissue or for storage^15^. While LPL is known to be expressed by parenchymal cells of a number of tissues including both white and brown adipose tissue, skeletal and cardiac muscles, brain, macrophages as well as some smooth muscles of large blood vessels in mammals^15,16^, knowledge about its cellular expression in chickens remains scarce. In the present study, we have demonstrated for the first time, that LPL is expressed by the vascular endothelium, especially in capillaries and small-caliber veins within the P. major muscles of chicken in both health (Legacy chickens and unaffected Ross birds) and disease (WB-affected Ross chickens) states.

The low LPL mRNA signal in arterial end accompanying a higher one in the capillary and venous endothelia in affected chickens may explain the occurrence of the artery-sparing lymphocytic phlebitis lesion in WB^1,3^. We speculate that the enhanced LPL expression in veins compared to arteries indicates elevated lipoprotein hydrolysis activity in the venous end of affected chickens. This in turn causes increased permeability of the venous endothelium, not only to free fatty acids (FFA) and lipoprotein remnants such as LDL following hydrolysis of VLDL^17^ and possibly portomicrons^18^ in the blood stream, but also to inflammatory cells including monocytes, lymphocytes and heterophils. The escaping immune cells contribute to the development of the lymphocytic phlebitis lesion. Indeed, this observation is in line with a previous study which showed a role of LPL in development of vascular diseases such as in atherogenesis^19^.

Evaluation of expression of lipid-related genes based on RNA-seq profile suggests an increase of lipid metabolism in affected chickens at week 3 of age. This is demonstrated by the upregulated genes whose functions include the following processes: uptake of lipids from circulating lipoprotein facilitated by LPL^15^, and to some extent, LIPG^20,21^; transportation of FFA across the plasma membrane into the cytoplasm of cells by CD36 for β-oxidation or re-esterification into triglycerides^22^; intracellular binding and shuttling of lipids and other hydrophobic molecules across different organelles within cells by FABP4^23^ and RBP7^24^, and storage of lipids by PLNI1^25^ and THRSP^26^. Other lipid-related biological functions that are likely occurring in affected chickens include elevated cholesterol metabolism as evidenced by the upregulation of ABCA1 which is involved in reverse cholesterol transport^27^, together with PLTP^28^ and LIPG, which encodes endothelial lipase^21^. It should be noted that both ABCA1 and PLTP were significantly upregulated in affected chickens at week 3 and week 7 of age, indicating that active cholesterol biosynthesis and transport accompany all stages of WB disorder. The current presentation agrees with recent studies in our laboratory that suggested increased cholesterol biosynthesis in the P. major muscles of feed-efficient chickens, which also show high susceptibility to WB^29,30^.

The RNA-seq expression profile of lipid-related genes in affected chickens at week 7 reveals statistically non-significant (FABP4, LIPG, LPL and RBP7) and significant downregulation (CD36, PLNI1 and THRSP), suggesting dysregulation of lipid metabolism between week 3 and week 7. While direct biological changes leading to this dysregulation is not apparent in the present study, other indirect causes such as hypoxia, which has been reported previously in WB-affected chickens^6,31^, could be playing a significant role in the aforementioned metabolic disruption. It has been widely established that hypoxia increases the expression of ANGPTL4, a potent inhibitor of LPL activity^32,33^. Another study showed that acute hypoxia decreases transcription of PPARγ, LPL and CD36 in white and brown adipose tissue^34^. Based on these observations, it is likely that hypoxia, starts earlier in life and increases gradually to reach a threshold when it affects other functional processes such as lipid metabolism. The upregulation of ANGPTL4 in affected chickens at week 7 in this study supports heightened hypoxia at market age. Additionally, hypoxia is implicated in increased cholesterol metabolism especially involving ABCA1 in macrophages^35^, which compares favorably with our findings at both week 3 and week 7 of age. Besides its effects on metabolism, hypoxia is also suspected to augment development of lymphocytic phlebitis that characterizes WB. This follows the role of hypoxia in activating vascular endothelial cells to increase their adhesiveness for leucocytes^36^, thereby allowing immune cells to cross the endothelial-cell barrier contributing to phlebitis.

The present study has also demonstrated presence of a consistent overexpression of slow-twitch myofiber-related genes in the P. major muscles of WB-affected chickens at week 3 and week 7 of age (market age). Even though the expression of ANKRD2, MYOM3, MYOZ2 and TNNI1 were not statistically significant in affected compared to unaffected chickens at week 3, they showed similar directionality as those genes whose expression were significantly upregulated in affected chickens at both age groups. This observation suggests that the onset and progression of WB in chickens is accompanied by expression of slow-twitch skeletal muscle genes, and that the expression increases with the severity of the disease as evidenced at week 7 of age. Corroborating the present findings, a study conducted by Kong *et al*.^37^, between unselected and genetically selected modern broiler chickens also showed upregulation of TNNI1, MYOZ2, MYBPC1, LMOD2 in the latter group, indicating a consistent molecular signature in the P. major muscles of modern broiler chickens.

Given that P. major muscles in chickens are almost purely type IIB muscle (glycolytic) fibers^9^, it follows, therefore, that the metabolism governing type II muscle fibers is different from that of type I (oxidative fibers)^12^. Hence, the appearance of slow-type proteins in a primarily glycolytic muscles such as P. major muscle would inevitably bring alterations in the overall muscle metabolism, especially relating to muscle bioenergetics. With the knowledge that the P. major muscles of modern broiler chickens have lower mitochondrial content with respect to their weight^38,39^, it is reasonable to suggest that the emergence of the slow-twitch muscle fibers would place undue bioenergetic demands to the muscle, especially in affected chickens. Indeed, skeletal muscles have been known to communicate their energy demands to other organs through paracrine and endocrine signaling mechanisms, effected by myokine secretions^40^. With this in mind, the emergence of slow-type muscle proteins in the P. major in the present study, would likely result in a shift or dysregulation of muscle metabolism, and therefore, adaptive responses with respect to muscle bioenergetics and myofibrillar structure taking effect. The upregulation of genes associated with increased lipid uptake and transport at week 3 of age in affected chickens, which coincide with the time when the expression of slow muscle genes begin to rise, serves to support changes in muscle metabolism towards oxidative phosphorylation. More specifically, MYBPCI, whose expression was upregulated in affected chickens at all time points in the current study, has been associated with intramuscular lipid deposition in beef cattle^41^. This observation also corroborates the findings of previous studies on WB which showed alterations of carbohydrate and lipid metabolism^5,8^. Additionally, our findings agree with a recent study which showed that increased expression of lipid metabolism genes in the P. major muscles of male broiler chickens as well as the cranial ends of the same muscle group was suggestive of metabolic switch and increased susceptibility to WB^42^.

Increasing expression of slow myofiber-type genes in affected muscles with disease severity may also reflect an adaptive response of the P. major muscle to biomechanical or overload stress as a result of a rapid increase in muscle weight over a short period. Fast-to-slow muscle transitions have been previously observed to be induced by stretch overload resulting in immobilization in a lengthened position^43,44^ and mechanical stress^45^. Along these lines, affected broiler chickens, which frequently have high-breast muscle yield/weight, due to elevated myofiber hypertrophy^46^ may be considered to have the greatest impact of biomechanical stress due to stretch overload. The low activity characterizing the behavior of modern broiler chickens further serves to support this argument. We therefore hypothesize that the rapid increase of pectoral muscle weight would inevitably impart stretch overload stress on individual myofibers resulting in immobilization in a lengthened position, thereby inducing the expression of slow myofiber genes in the predominantly glycolytic muscle. This is especially true for myofibers located superficially on angulated or curved regions of the pectoral muscles (e.g. cranial region), which are frequently affected first in comparison to those located in deeper regions.

In the case of muscle overload stress, it would be expected that some of the “first responder genes” will be those associated with mechanosensing, whose role would be to bring about muscle adaptability and homeostasis. Accordingly, several genes that were upregulated in affected chickens in the current study have been linked with mechanosensing and enhancement of structural support in muscles. CSRP3 gene, which encodes muscle LIM protein (MLP)^47^, and found in the sarcolemma, costameres and the sarcomere of muscles where it binds specific proteins of the myofiber in the aforementioned regions^48^, has been shown to play a role in mechano-signaling processes^49,50^. CSRP3’s role of mechanosensing and structural support in the current study is evidenced by the localization of the CSRP3 mRNA near the sarcolemma and probably costameres of myofibers where the protein product (MLP) is thought to confer its maximal mechanosensory effect in response to external mechanical stress. Further, the focal expression pattern of CSRP3 in areas of myofibers that are contiguous with large blood vessels in unaffected chickens suggests a response to external mechanical stimulus. It is, therefore, not surprising that CSRP3 had the highest expression at both time points, especially at week 7 (Log2 FC 6.2) coinciding with the severe degree of pathology of the muscles possibly due to increased fibrosis from WB.

Another slow myofiber gene associated with myofibrillar support is MYBPC1, which encodes the myosin-binding protein C, slow skeletal isoform (MyBP-C)^51^ that binds to myosin and titin thereby conferring stability and maintenance of the sarcomeric A-band^52^. Therefore, increased expression of MYBPC1 in affected chickens, coupled with homogenous distribution of its mRNA in myofibers further affirms its role in maintaining muscle integrity, especially in the face of WB. Indeed, the association of MYBPC1 with progression of muscular dystrophy in chickens^53^ and mice^54^, and WB in the current study suggest the importance of this gene in development of myopathies. Taken together, these observations demonstrate the role of MYBPC1 gene as a potential biomarker for WB progression in meat-type chickens.

ANKRD2, which belongs to the members of the ankyrin repeat protein, and known to interact with titin at the sarcomeric I-band, is highly expressed in slow-twitch compared with fast-twitch muscles^50,55^. Through its mechanosensory action, the expression of ANKRD2 in oxidative striated muscles has been associated with a response to increased mechanical stress thereby contributing to maintenance of structural stability of muscles^50^. While the same function has not been reported for type II muscle fibers, it is possible that the upregulation of ANKRD2 in the P. major muscles of affected chickens in the current study could be a response to increased mechanical stress emanating from individual myofibers that are under continuous hypertrophy, fibrosis or weight overload as the pathology of WB progresses.

Leiomodin 2 is a protein that belongs to a class of potent tandem-G-actin binding nucleators that promote actin polymerization in muscles^56^. In promoting actin nucleation, leiomodin proteins aid in lengthening, assembly, maintenance of actin/thin filament lattice structure in muscles as well as muscle contraction^57^. LMOD2 functions primarily as an actin nucleator in the cardiac muscles^58^, and to a lesser extent, in mammalian adult skeletal muscles^57,59^. Therefore, the upregulation of LMOD2 in the present study is in line with increased muscle fiber-type remodeling in WB-affected chickens specifically relating to actin filament functions. As an actin-binding protein, LMOD2 is thought to act in concert with other slow myofiber-type proteins in imparting structural support and functionality within the P. major muscles. It is worth noting that the expression of LMOD2 in affected chickens increases from week 3 to week 7 suggesting an association with the progression of the WB myopathy.

Myomesin 3 protein encoded by the MYOM3 gene, belongs to myomesin family that localize in the M-band of the sarcomere^12,60^. Myomesin proteins solely found in striated muscles, are involved in binding of myosin to other proteins such as titin thereby stabilizing the thick filament lattice and the sarcomere structure in general during periods of sustained mechanical loading^60,61^. MYOM3 was found to be highly expressed in slow muscles^60^. In the present study, the upregulation of MYOM3 alongside other slow-type muscle genes in the P. major muscles of affected chickens at week 7 of age provides evidence of the association of slow myofiber-phenotype with WB disease process. Given that the severe form of the WB myopathy frequently occurs around market age (week 7 of age), it is likely that the upregulation of MYOM3 in affected chickens at this stage, serves to augment the structural support of the remaining healthy myofibers in the face of the WB disease. This, therefore, is in line with the adaptive response of the P. major muscles to increased mechanical loading of the breast muscle that is complexed with WB condition.

Myozenin 2 protein, also called calsarcin 1 or FATZ 2 (filamin-, actinin-, and telethonin-binding protein of the Z-disc), and encoded by the MYOZ2 gene, is localized exclusively in the Z-disc of muscles. MYOZ2 gene is expressed in cardiac muscles and slow-twitch skeletal muscles of mammalian adults and developing embryos^12,62^. Myozenin primarily binds calcineurin/calmodulin and other proteins in the Z-disc such as alpha-actinin, telethonin and myotilin^12,62^, which in the present case, would bring about structural stability of the muscle sarcomere during the progression of WB in chickens. Lastly, TNNI1, which encodes inhibitory troponin I of the troponin protein subunits in slow-skeletal muscles^63^, has been demonstrated to be involved in myofiber switches in response to changes in functional demands in muscle such as mechanical unloading^64^. This agrees with our current observation to a certain extent, where the late stage of WB is associated with increased myodegeneration and myonecrosis, and hence, the remaining functional myofibers would be subjected to increased workload.

Additionally, while it was previously thought that the expression of slow-type myofiber genes in P. major muscles of WB-affected chickens was due to myoregeneration^6^, the current study clarifies that it is not the case. This is exemplified by the expression of CSRP3 and MYBPC1 genes in mature myofibers of affected chickens in the present study.

In conclusion, this is the first study to show the expression of LPL by the vascular endothelium in chickens. This study also provides mechanistic insights into the alteration of lipid metabolism in the course of WB disease process. Further, the present study confirms the occurrence of slow myofiber phenotype during the progression of WB in chickens and its role in skeletal muscle remodeling. The association of some slow myofiber genes such as MYBPC1 with muscle dystrophies, which are known to occur due to genetic defects, also suggests a possibility of genetic alterations affecting gene expression or protein functions in WB.

## Materials and Methods

### Ethics statement

The animals (Ross 708 and Legacy chickens), and experiments used in this study were approved by the University of Delaware’s Institutional Animal Care and Use Committee (IACUC) under protocol number 72R-2017-0. Further, birds were raised in strict adherence to the guidelines provided by the Agricultural animal care and use in research and teaching handbook of the College of Agriculture and Natural Resources, University of Delaware.

### Experimental birds and muscle sample processing for RNA *in situ* hybridization

The chickens used for RNA *in situ* hybridization in this study were from two sources namely Ross 708 and Legacy birds, also referred to as Heritage chickens (from the University of Illinois). The Legacy chickens were used in this experiment as a baseline group for RNA *in situ* hybridization protocol on the selected genes. Legacy chickens have not been subjected to genetic selection for fast-growth rate and high muscle yield since 1950s, in contrast to Ross birds which are modern commercial broiler chickens genetically selected for fast-growth rate, high feed efficiency and breast muscle yield^65^. Consequently, the Legacy chickens do not develop WB myopathy and have no history of the myopathy so far. Commercial broiler chickens such as Ross birds, on the other hand, are known to be susceptible to WB, and have been reported to develop the myopathy^1^.

Eggs from the respective chicken types were incubated and hatched 2 days apart at the University of Delaware; the eggs of Ross birds were the first to hatch. The difference in day of hatching was occasioned by a 2-day delay in acquisition of eggs of Legacy birds. All the chickens were raised in the same pen within the chicken houses at the University of Delaware from June to July 2018. The birds were allowed free access to water and feed until they were euthanized. Throughout the experimental period, the birds were provided with standard commercially formulated feed for broiler chickens. This study utilized chickens at day18 post-hatch for Legacy (n = 7) and at day 20 post-hatch for Ross birds (n = 8). The chickens were euthanized by cervical dislocation and pectoral major muscle samples harvested from the cranial aspect of the right pectoral region and immediately fixed by immersion into 10% neutral buffered formalin until further processing for microscopic analysis.

To identify suitable samples for RNA *in situ* hybridization, all harvested samples were first processed routinely for histological examination with Hematoxylin & Eosin (H/E) as outlined in a previous study^3^. Briefly, muscle tissue samples were trimmed, paraffin-embedded into blocks, sectioned 4–5 μm thickness and stained with H/E at the University of Delaware comparative pathology laboratory (Newark, DE). Both transverse and longitudinal sections of the P. major muscles were prepared and subsequently examined using an Olympus BX40 light microscope. Microscopic analysis of the muscle samples entailed examination for presence of tissue pathology associated with WB. For tissues with WB pathology, the tissue slides were scored to indicate the extent of tissue damage using the parameters applied in a previous study^3^. Therefore, a subset of samples comprising unaffected Ross birds (n = 3), moderately affected chickens (n = 3) and Legacy chickens (n = 3) were identified and used for RNA i*n situ* hybridization. Since all Legacy chickens were scored as unaffected, 3 samples were selected randomly from the rest to match those of the Ross groups. The respective paraffin embedded tissue blocks of the selected samples were used for RNA *in situ* hybridization.

### RNA *in situ* hybridization protocol

RNA *in situ* hybridization was performed using the RNAscope 2.5 assay for formalin-fixed paraffin-embedded (FFPE) protocols (Advanced Cell Diagnostics (ACD) Inc., Gateway, CA) according to manufacturer’s instructions. Firstly, the samples were subjected to pretreatment protocol based on document number 322452 USM (ACD), while hybridization, amplification and target detection protocols used were based on document 322500-USM for RNAscope 2.5 HD Duplex detection kit (ACD). Briefly, 4–5 µm formalin-fixed and paraffin-embedded pectoral muscle tissue sections were obtained from the selected paraffin embedded tissue blocks, pretreated with heat at 60 °C for 1 hour, cleared in xylene and dehydrated in 100% EtOH followed by target retrieval for 30 min and then protease treatment for 30 minutes prior to hybridization.

This protocol targeted four genes in the pectoral muscles whose probes were prior developed by ACD. The probes for the target genes included lipoprotein lipase (LPL), Entrez ID 396219 targeting region 199–1306 of NM_205282.1 (cat No. 558349); perilipin 1 (PLIN1) Entrez ID 415487, targeting region 174–1143 of NM_001199486.1 (cat No. 558369-C2); Myosin binding protein C1 (MYBPC1) Entrez ID 418099, targeting region 118–1147 of XM_025155759.1 (Cat No. 558359) and cysteine and glycine rich protein 3 (CSRP3) Entrez ID 174–1143 of NM_001199486.1 (cat No. 558181-C2). To allow for examination of expression of two related genes concurrently, the four probes were hybridized in pairs. Hence, the two pairs of genes examined included lipid-related genes (LPL and PLIN1) and muscle-related genes (MYBPC1 and CSRP3).

Running parallel to target probes were positive and negative control probes. The positive control probes used were Gg-UBC-C2 (cat No. 453961-C2), detected by the red signal, and Gg-PPIB (cat No. 453371), detected by the green signal all performed in duplex. RNAscope 2-plex negative control probe (cat No. 320751) was used as the negative control. To examine all target and control probes in this study, 4 tissue sections were obtained from each of the selected samples followed by processing for lipid-related genes, muscle-related genes, positive control probes and negative control probes. Hence, all the probes were hybridized to all selected sample sections for 2 hours at 40 °C in a HybEZ oven (ACD), washed with 1X wash buffer and stored in 5X SSC solution overnight until the following day. On the second day, all the samples were subjected to amplification steps as well as signal detection as per the protocol. For signal detection, the red signal targeted the mRNAs of PLIN1 and CSRP3 genes while the green signal targeted the mRNAs of LPL and MYBPC1 genes. The tissue slides were then counter-stained with Gill’s Hematoxylin II stain and subsequently examined using an Olympus BX40 light microscope and relevant photomicrographs collected using a Nikon DS-Fi2 camera and NIS Elements D software.

### RNA-sequencing data of muscle-related and lipid-related genes

Besides RNA *in situ* hybridization, this study also used RNA-seq data of specific genes related to lipid metabolism and slow-myofiber phenotype frequently associated with WB myopathy. The genes were identified and selected from RNA-seq data in our laboratory. The RNA-seq data were derived from the P. major muscles belonging to 2 distinct high-breast-muscle-yield, purebred chicken broiler lines at 3 weeks of age^7^ and another one at 7 weeks of age^6^.

The protocol used in processing of the P. major muscle samples for RNA-seq were previously documented for chickens at 3 weeks of age^7^, and 7 weeks of age^6^. Of note is that the two RNA-seq datasets (week 3 and week 7) were sequenced using Illumina sequencing platform for short reads^6,7^. To use the newer version of the software packages and the most recent chicken reference genome (Ensemble Gallgall 6.0 and gene annotation file release 95), raw RNA-sequencing reads for both week 3 and week 7 muscle samples were reanalyzed using the following steps: (1) sequence reads were quality-checked and quality-trimmed using FastQC v0.11.7 and trimmomatic v0.38, respectively; (2) the trimmed sequence reads were mapped to the chicken reference genome using Hisat2 v2.1.0; (3) SAM files were converted to BAM files using samtools v1.9; (4) geometric normalization of library sizes and identification of differentially expressed genes (FDR-adjusted p-value < 0.05) were determined using Cuffdiff v2.2.1, a software tool within Cufflinks package^66,67^. The Cuffdiff software estimates gene expression abundance from aligned reads in two or more samples and tests the statistical significance of each observed change in expression between the conditions, by application of a beta negative binomial distribution model at gene- and transcript-level resolution^67^. In the present study, the two conditions were unaffected and WB-affected chicken samples with several biological replicates in each case. The Cuffdiff software also uses statistical modeling to account for variability in gene and transcript measurements across biological replicates^66,67^. We used the default pooled dispersion method to model for cross-replicate dispersion estimation in Cuffdiff. The Cuffdiff software identifies a differentially expressed gene or transcript by testing the observed log fold-change in its expression against the null hypothesis of no change and assesses the significance of each comparison at (FDR-adjusted p-value < 0.05)^66^. While the Cuffdiff software produces several output result-files, the focus of the present study was on the gene-level differential expression file, which is generated by examining the test differences in the summed ﻿Fragments Per Kilobase of transcript per Million mapped reads (FPKM) values of transcripts sharing each gene identity between unaffected and affected conditions.

In addition, new adjustments incorporated during the processing of sample data at week 7 of age included use of 5 unaffected and 5 affected samples instead of 6 unaffected and 5 affected samples used in the previous study^6^. This was because one unaffected sample (sample 51 unaffected) displayed molecular characteristic consistent to those of affected muscle samples and was believed to be misclassified, as suggested by the authors^6^, hence was excluded from the current analysis.

From the RNA-seq data of the two chicken lines, the expression profile involving Log2 fold-change (Log2FC) of the selected genes between WB-affected and unaffected birds at each of the 2-time points was extracted. A time comparison of the lipid-related genes and muscle-related genes between week 3 and week 7 of age for each gene set was made. The lipid-related genes examined included ATP-binding cassette sub-family A1 (ABC1), angiopoietin-like factor 4 (ANGPTL4), fatty acid translocase (CD36), fatty acid binding protein 4 (FABP4), lipase G (LIPG), lipoprotein lipase (LPL), perilipin 1 (PLIN1), phospholipid transfer protein (PLTP), retinol binding protein 7 (RBP7) and thyroid hormone responsive (THRSP). Muscle-related genes examined included ankyrin repeat domain 2 (ANKRD2), cysteine and glycine rich protein 3 (CSRP3), leiomodin 2 (LMOD2), myosin binding protein C, slow type (MYBPC1), myomesin 3 (MYOM3), myozenin 2 (MYOZ2), and troponin I1, slow skeletal type (TNNI1).

## Data Availability

Raw RNA-sequencing data of chicken P. major muscle samples is available at NCBI Sequence Read Archive (SRA study accessions: SRP150752 for samples at week 3 of age; SRP224368 for samples at week 7 of age).
